# *SMURF1* Downregulation Highlights Its Potential Role in Breast Cancer

**DOI:** 10.3390/ijms27041921

**Published:** 2026-02-17

**Authors:** Leyla Tekin, Funda Dinç, Cenk Yazkan, Murat Cenik, Çilem Özdemir, Onur Amaç, Tuba Edgünlü

**Affiliations:** 1 Department of Pathology, Faculty of Medicine, Muğla Sıtkı Koçman University, Muğla 48000, Turkey; onuramac@mu.edu.tr; 2 Department of Radiology, Faculty of Medicine, Muğla Sıtkı Koçman University, Muğla 48000, Turkey; fundadinc@mu.edu.tr; 3 Department of Surgical Medical Sciences, Faculty of Medicine, Muğla Sıtkı Koçman University, Muğla 48000, Turkey; cenkyazkan@mu.edu.tr; 4 Department of Medical Biology, Institute of Health Sciences, Muğla Sıtkı Koçman University, Muğla 48000, Turkey; muratcenik5858@gmail.com; 5 Department of Bioinformatics, Graduate School of Natural and Applied Sciences, Muğla Sıtkı Koçman University, Muğla 48000, Turkey; cilemmozdemir@gmail.com; 6 Department of Medical Biology, Faculty of Medicine, Muğla Sıtkı Koçman University, Muğla 48000, Turkey

**Keywords:** breast cancer, *SMURF1*, *SMURF2*

## Abstract

This study aimed to evaluate the mRNA and protein levels of *SMURF1* and *SMURF2* in breast cancer and to elucidate their potential biological roles through in silico analyses. Tumor and adjacent normal tissue samples were collected from 30 newly diagnosed breast cancer patients who underwent mastectomy. The mRNA expression levels of *SMURF1* and *SMURF2* were analyzed by quantitative PCR (qPCR), and their protein expression patterns were evaluated using immunohistochemistry (IHC). In addition, protein–protein interaction (PPI) and functional enrichment analyses were performed via the STRING database to identify potential molecular interactions and biological pathways associated with these genes. The mRNA expression level of *SMURF1* was significantly downregulated in tumor tissues compared to normal breast tissues (*p* = 0.002), whereas no significant difference was observed in *SMURF2* mRNA expression (*p* = 0.981). IHC results revealed that *SMURF1* and *SMURF2* protein levels did not differ significantly between tumor and normal samples. The in silico analysis demonstrated that *SMURF1* and *SMURF2* interact with multiple proteins involved in key signaling pathways, particularly the TGF-β/BMP and Wnt/β-catenin pathways. The findings suggest that the downregulation of *SMURF1* in breast cancer may contribute to tumor progression by enhancing Wnt/β-catenin signaling activity. The interactions of *SMURF1* and *SMURF2* with TGF-β/BMP pathway regulators indicate that these genes may play dual roles in both tumor-suppressive and oncogenic mechanisms, depending on the cellular context.

## 1. Introduction

Breast cancer accounts for approximately 12% of all cancer types worldwide and 25% of cancer diagnoses in women. Due to its heterogeneous nature, breast cancer poses clinical challenges in diagnosis and treatment; breast tumors display diverse morphologies and molecular characteristics [1]. Transforming growth factor-beta (TGF-β) is a dynamic growth factor that regulates various cellular functions and serves as a key regulator in normal mammary gland development as well as in cancer progression and metastasis [2]. In cancer pathogenesis, the activation or inhibition of the TGF-β pathway is associated with carcinogenic processes such as epithelial–mesenchymal transition (EMT), tumor cell resistance to chemotherapy, metastasis, cancer stem cell behavior and angiogenesis [3,4].

*SMURF1* and *SMURF2* (Smad Ubiquitin Regulatory Factors 1 and 2) are negative regulators of the BMP/TGF-β pathway and play roles in tissue homeostasis, embryogenesis, and various human diseases, including cancer. These proteins mediate the proteasomal degradation or autophagic removal of target proteins by tagging them with ubiquitin [5]. *SMURF1*, an E3 ubiquitin ligase, regulates intracellular protein degradation and predominantly exhibits oncogenic properties. In cancer, *SMURF1* promotes lamellipodia formation, cell migration, and invasion by modulating RhoA/ROCK/MLC2 signaling [6]. Overexpression of *SMURF1* in gastric cancer has been associated with increased cell motility, invasion, and poor clinical prognosis [7]. At the level of hormonal signaling, *SMURF1* interacts with estrogen receptor alpha (ERα) and androgen receptor (AR), enhancing their stability and thereby promoting tumor growth in breast and prostate cancers [8,9].

*SMURF2*, which shares a similar structure with *SMURF1*, is also an E3 ubiquitin ligase that exerts dual roles—both tumor-suppressive and oncogenic—depending on the cellular context. In normal cells, *SMURF2* maintains genomic integrity by participating in DNA damage response, chromatin organization, and cell cycle regulation. It ensures genetic stability and prevents tumor formation by targeting proteins such as RNF20 and Topo IIα [10,11]. Moreover, *SMURF2* limits cell proliferation by degrading oncogenic transcription factors including KLF5, YY1, ID1/3, and EZH2 [12,13,14]. However, in certain cancer types, *SMURF2* expression is upregulated, leading to activation of signaling pathways such as EGFR, RAS and Wnt/β-catenin which promote cell growth, drug resistance and metastasis [5]. Overexpression of *SMURF2* has been detected in esophageal squamous cell carcinoma and chemoresistant hepatocellular carcinoma [15,16]. In breast cancer cells, suppression of *SMURF2* has been shown to enhance in vitro cell migration and in vivo bone metastasis [17].

Although the involvement of *SMURF1* and *SMURF2* in the progression of various cancers such as colorectal, lung, and gastric cancers has been well-documented, comprehensive investigation into their combined roles and clinical implications specifically in breast cancer remains limited [5,7]. Given their critical regulatory functions in other malignancies, there is a clear need to elucidate their expression patterns and potential as prognostic biomarkers in breast cancer. This study aims to address this gap by providing a thorough analysis of *SMURF1* and *SMURF2*, highlighting their importance in the molecular landscape of breast cancer.

The aim of this study was to determine the expression levels of *SMURF1* and *SMURF2* genes in breast cancer at both the mRNA and protein levels and to elucidate their potential biological roles. For this purpose, the mRNA expression levels of *SMURF1* and *SMURF2* were analyzed using quantitative PCR (qPCR), while their protein expression patterns were determined by immunohistochemistry (IHC). In addition, PPI network and functional enrichment analyses were performed using the STRING database to explore the potential molecular interactions and biological pathways associated with these genes.

## 2. Results

### 2.1. Clinic and Pathological Profile of Cases

Among the study cohort, 15 patients (50%) exhibited a Ki67 index of 14% or higher, while 11 patients had tumors smaller than 2 cm. Seven patients were younger than 50 years, and 23 patients were 50 years or older. The lymphovascular invasion (LVI) was observed in 16 patients, and the perineural invasion (PNI) was present in 11 patients. Two patients were classified as triple-negative (Table 1).

### 2.2. Immunohistochemical Analysis

Immunohistochemical evaluation of ER (clone 6F11, 1:50; Leica Biosystems, Nussloch, Germany, Thermo Fisher Scientific, Waltham, MA, USA), PR (clone Pgr16, 1:100; Leica Biosystems), Ki67 (clone MM1-optimized; Leica Biosystems), cerbB2 (clone 10A7, 1:40; Leica Biosystems), the *SMURF1* antibody (1:100; Proteintech, polyclonal, Manchester, UK) and the *SMURF2* antibody (1:600; Proteintech, polyclonal, Manchester, UK) was performed using the Leica Bond-Max automated staining system. Immunohistochemical staining was evaluated using a light microscope (BX46 Clinical Microscope, Olympus, Tokyo, Japan).

**Figure 1:**
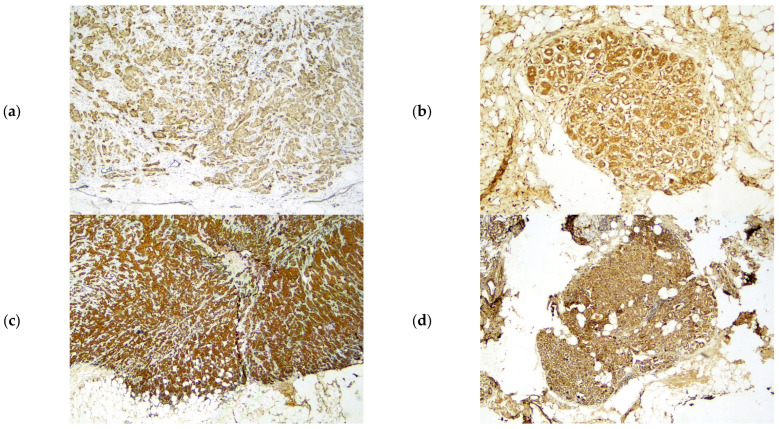
Immunohistochemical Expression of *SMURF1* and *SMURF2* in Breast Tumor and Normal Tissues. Immunohistochemical staining of *SMURF1* (**a**) and *SMURF2* (**c**) proteins in breast tumor tissue, and *SMURF1* (**b**) and *SMURF2* (**d**) in normal breast tissue (4× magnification). (scale bar = 50 µm). Brown staining indicates positive immunohistochemical expression.

PR and ER expression levels were assessed using the Allred scoring system, in which staining intensity in positively stained tumor nuclei was graded as 0 (no staining), 1 (weak), 2 (moderate), or 3 (strong). CerbB2 status was interpreted as negative for scores of 0 or 1+, and positive for scores of 3+, while cases with a 2+ score were further analyzed using SISH for confirmation. ER and PR positivity was defined as nuclear staining in more than 1% of tumor cells. For Ki67, it was defined as high and low staining based on 14% of tumor cells. *SMURF1* and *SMURF2* immunoreactivity was evaluated semiquantitatively based on staining intensity, scored from 0 to 3 (0 = absent, 1 = weak, 2 = moderate, 3 = strong) [18] (Figure 1). *SMURF1* and *SMURF2* immunoreactivity predominantly displayed cytoplasmic staining in both tumor and adjacent normal tissues. The intensity and proportion of positive cells were evaluated semi-quantitatively on a four-tiered scale: 0 = absent, 1 = weak (≤10%), 2 = moderate (11–50%), 3 = strong (>50%). Representative examples of each positivity level and all patient scores are listed in Table 1. Scale bars (50 µm) were added to Figure 1.

### 2.3. Expression Levels of SMURF1 and SMURF2

**Figure 2:**
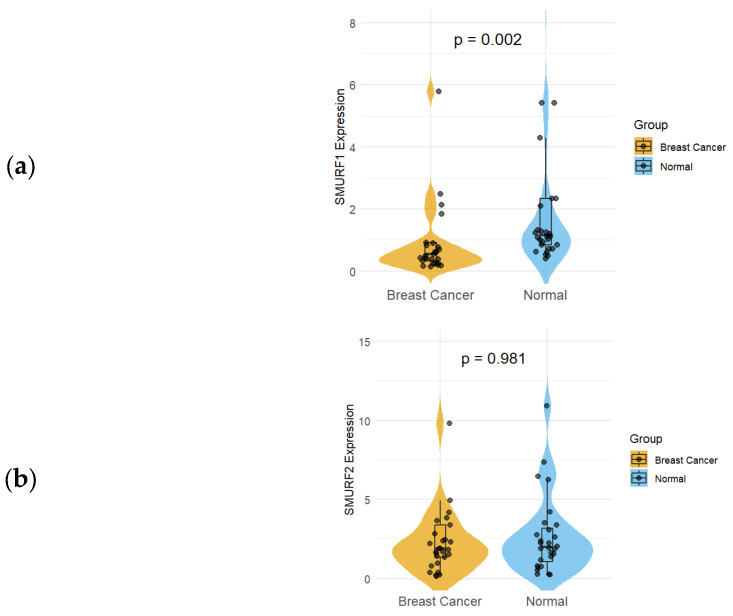
Expression levels of (**a**) *SMURF1* and (**b**) *SMURF2* between tumor and normal tissues.

Tumor and normal tissue samples from breast cancer patients were stained with anti-*SMURF1* and anti-*SMURF2* antibodies to evaluate differences in *SMURF1* and *SMURF2* expression using immunohistochemistry. The results showed that *SMURF1* and *SMURF2* protein expression levels did not differ significantly between tumor and normal tissues (*p* > 0.05). However, when *SMURF1* and *SMURF2* mRNA expression levels were compared, *SMURF1* was found to be significantly reduced in tumor tissues (*p* = 0.002), whereas *SMURF2* expression did not differ significantly between the groups (*p* = 0.981) (Table 2) (Figure 2).

### 2.4. In Silico Validation of Experimental Findings

We constructed PPI networks based on *SMURF1* and *SMURF2* interactions at three hierarchical levels (Table 3). The first-level network, which included *SMURF1* and *SMURF2*, showed a significant interaction (*p* = 0.0193). Gene Ontology (GO) enrichment revealed that “ubiquitin-dependent SMAD protein catabolic process” (GO:0030579) and “Wnt signaling pathway” (GO:0060071) were the most prominent cellular processes. KEGG pathway analysis revealed enrichment of “Hedgehog signaling pathway” (hsa04340). The second-level network consists of 11 proteins, including *SMURF1*, *SMURF2*, SMAD5, RUNX2, SMAD1, SMAD6, SMAD7, SMAD2, TGFBR1, TGFBR2, and RHOA. GO analysis highlighted the “transmembrane receptor protein serine/threonine kinase signaling pathway” (GO:0007178) and the “BMP signaling pathway” (GO:0030509) as significantly enriched processes. KEGG analysis revealed the “TGF-beta signaling pathway” (hsa04350) as the most enriched pathway. Similarly, in the third-level network, which includes *SMURF2*, *SMURF1*, SMAD3, SMAD6, SMAD2, TGFBR1, USP15, UBC, SMAD7, RPS27A, and UBE2L3, GO terms such as “Transmembrane receptor protein serine/threonine kinase signaling pathway” and “BMP signaling pathway” were enriched, and KEGG analysis again showed enrichment of “TGF-beta signaling pathway.” These results indicate that *SMURF1* and *SMURF2* interact with multiple proteins involved in important signaling pathways, particularly those related to the TGF-beta and BMP signaling pathways, highlighting their potential roles in regulating cellular signaling processes. To further substantiate our results, we performed an additional in silico validation using the TCGA breast cancer (BRCA) dataset via the GEPIA2 web server (http://gepia2.cancer-pku.cn/ accessed on 8 May 2025). Consistent with our qPCR data, *SMURF1* mRNA showed significantly lower expression levels in tumor tissues compared with matched normal samples (*p* < 0.01), while *SMURF2* expression remained unchanged. These findings provide independent support for our experimental observations.

#### 2.4.1. Prognostic Value and Patient Survival Analysis

**Figure 3:**
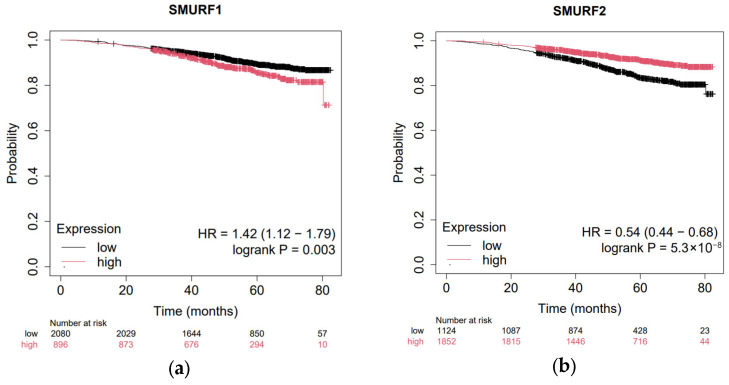
Patient stratification was performed using the “auto-select best cutoff” feature, which explains the unequal distribution of patients in high- and low-expression groups (*n* values), aimed at identifying the most clinically significant prognostic impact. Kaplan–Meier survival analysis of breast cancer patients stratified by SMURF gene expression. (**a**) Patients were divided into high- and low-expression groups based on the median expression of *SMURF1* using the Kaplan–Meier Plotter platform. High *SMURF1* expression was significantly associated with poorer overall survival. (**b**) Kaplan–Meier survival analysis of patients stratified by *SMURF2* expression. No significant association between *SMURF2* expression levels and overall survival was observed. Hazard ratios (HRs), 95% confidence intervals (CIs), and log-rank *p*-values are shown.

Kaplan–Meier survival analysis revealed that high *SMURF1* expression was significantly associated with poorer overall survival in breast cancer patients, indicating an adverse prognostic impact. In contrast, *SMURF2* expression did not show a significant association with overall survival, suggesting distinct clinical roles for these two SMURF family members (Figure 3).

#### 2.4.2. Differential Expression of *SMURF1* and *SMURF2* in Breast Cancer Tissues

Analysis of TCGA-BRCA and GTEx datasets using GEPIA3 demonstrated that *SMURF1* expression was significantly altered in breast cancer tissues compared to normal breast samples (*p* = 2.89 × 10^−19^). Conversely, *SMURF2* did not exhibit a significant differential expression pattern (*p* = 0.238), further supporting the notion that *SMURF1* may play a more prominent role in breast cancer biology.

#### 2.4.3. Single-Cell RNA Sequencing Revealed Cell-Type-Specific Expression of SMURF Genes

**Figure 4:**
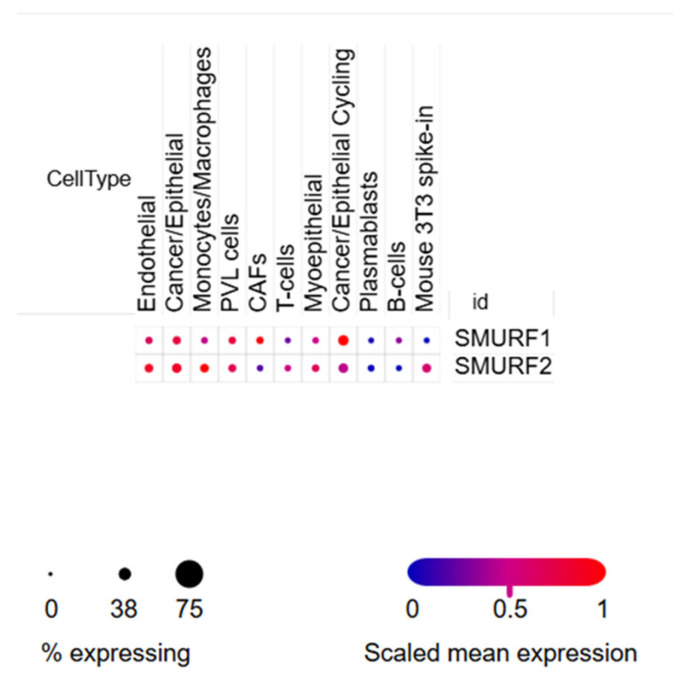
Cell-type-specific expression of *SMURF1* and *SMURF2* in breast cancer assessed by single-cell RNA sequencing. Dot plots show *SMURF1* and *SMURF2* expression across major cellular compartments within breast tumors, including malignant epithelial cells, stromal cells, and immune populations.

Single-cell RNA sequencing (scRNA-seq) analyses showed that both genes clustered in different cell populations within the tumor microenvironment. The highest expression density and prevalence were detected in the “Cancer/Epithelial Cycling” cell group. Moderate expression was also observed in CAFs, endothelial, and PVL cells. Compared to *SMURF1*, it exhibited a more widespread distribution, being highly expressed in endothelial, cancer/epithelial, monocyte/macrophage, and PVL cells (Figure 4).

## 3. Discussion

In this study, we investigated the expression patterns of *SMURF1* and *SMURF2* in breast cancer tissues and their adjacent normal counterparts, as well as their potential molecular interactions and functional implications, through in silico analyses. Our findings revealed a significant downregulation of *SMURF1* mRNA levels in tumor tissues compared to normal breast tissues, while *SMURF2* expression did not show a statistically significant difference. Immunohistochemical analysis indicated no significant difference in *SMURF1* and *SMURF2* protein levels between tumor and normal samples. Furthermore, the in silico functional enrichment analysis demonstrated that both *SMURF1* and *SMURF2* are strongly associated with the TGF-β, BMP, and Wnt signaling pathways, supporting their involvement in oncogenic and tumor-suppressive processes.

A notable finding in our study is the apparent paradox between the downregulation of *SMURF1* mRNA in our clinical cohort and its association with poorer prognosis at higher expression levels in the in silico survival analysis. This observation highlights the context-dependent dual role of *SMURF1* in breast cancer. While *SMURF1* downregulation might be an early event in tumorigenesis to bypass certain regulatory checkpoints (such as the inhibition of Wnt signaling), its relative maintenance or later upregulation in established tumors could drive aggressive behavior. High *SMURF1* levels have been shown to promote epithelial-to-mesenchymal transition (EMT) and metastasis through the degradation of RhoA. Therefore, while typically reduced in tumor tissues compared to normal, patients who retain higher relative *SMURF1* levels within the tumor population may face a higher risk of disease progression.

Regarding *SMURF2*, despite its strong prognostic significance (HR = 0.54), we observed no significant differential expression between tumor and normal tissues. This suggests that *SMURF2* may not serve as a primary diagnostic marker for malignancy in breast tissue, but rather functions as a crucial stratification marker once the tumor is established. The stability of *SMURF2* levels in our cohort, combined with its protective effect in survival datasets, indicates that its biological impact is likely mediated through its functional activity on specific substrates rather than gross changes in transcript levels.

Interestingly, despite its significant prognostic impact (HR = 0.54) observed in silico, *SMURF2* did not show differential expression in our clinical cohort. This suggests that *SMURF2* may function more as a predictive marker for disease progression rather than a primary diagnostic marker for malignancy. The stable expression of *SMURF2* across tumor and normal tissues further supports its complex, context-dependent role, where its biological impact is likely mediated through the selective ubiquitination of specific oncogenic or tumor-suppressive substrates rather than gross changes in its transcript levels.

Although *SMURF1* has predominantly been characterized as an oncogenic E3 ubiquitin ligase promoting cell migration and invasion, emerging evidence suggests that it may exert tumor-suppressive functions under specific cellular contexts. Recent studies have revealed that *SMURF1* negatively regulates several oncogenic pathways through selective ubiquitination of cancer-promoting substrates. *SMURF1* targets TRIB2, a positive regulator of hepatocellular carcinoma (HCC) survival and transformation, for ubiquitin-mediated degradation following its phosphorylation by p70S6K, thereby limiting TRIB2-driven tumorigenesis [19]. Moreover, *SMURF1* acts as a scaffold component in a complex with COP1 and β-TrCP, suppressing TCF4/β-catenin signaling and reducing oncogenic transcriptional activity in liver cancer cells [20]. Another important tumor-suppressive mechanism of *SMURF1* involves the AXIN1–Wnt signaling axis. *SMURF1* promotes non-proteolytic Lys29-linked polyubiquitination of AXIN1, preventing its binding to the Wnt co-receptor LRP5/6, thereby inhibiting Wnt-induced LRP6 phosphorylation and subsequent pathway activation [21]. Since aberrant activation of Wnt/β-catenin signaling is a hallmark of aggressive breast cancer subtypes, the reduced *SMURF1* expression observed in our study may lead to a loss of this negative regulation, potentially contributing to enhanced Wnt activity and tumor progression.

*SMURF1* has also been reported to mediate the degradation of MCAM (melanoma cell adhesion molecule), which drives proliferation, metastasis, and angiogenesis in HCC cells [22]. By promoting MCAM degradation in cooperation with β-TrCP, *SMURF1* may counteract pro-metastatic processes. Furthermore, *SMURF1* induces ubiquitination of the splicing factor SRSF5, a pro-oncogenic regulator of alternative splicing that favors tumor growth [23]. *SMURF1*-dependent degradation of SRSF5 leads to the accumulation of the tumor-suppressive isoform CCAR1L, thereby inhibiting proliferation and promoting cell-cycle arrest [24].

Recent studies have further elucidated the complex role of the TGF-beta pathway in the tumor microenvironment of solid tumors. It is now understood that *SMURF1* and *SMURF2* mediate the ubiquitination and degradation of TGF-beta receptors and SMAD proteins, acting as molecular switches that control the transition of TGF-beta from a tumor suppressor to a pro-metastatic factor. Particularly in breast cancer, the dysregulation of this pathway is closely linked to epithelial–mesenchymal transition (EMT) and chemoresistance [25].

In our study, we observed a significant downregulation of *SMURF1* mRNA levels in tumor tissues, whereas *SMURF1* protein expression remained relatively unchanged. This apparent discrepancy between mRNA and protein levels is a well-recognized phenomenon in cancer biology. Recent large-scale proteogenomic studies have shown that the correlation between mRNA and protein abundance is often modest, typically ranging from 0.40 to 0.60, as protein levels are heavily influenced by translational regulation and post-translational stability. Specifically, *SMURF1* is an E3 ubiquitin ligase that can undergo self-ubiquitination and proteasomal degradation. The maintenance of *SMURF1* protein levels despite decreased mRNA transcripts might suggest an increased protein half-life or a compensatory reduction in its degradation rate within the tumor microenvironment. Our findings are consistent with the TCGA-BRCA dataset, which also demonstrates significant mRNA downregulation, highlighting the complex regulatory landscape of *SMURF1* in breast cancer.

The in silico PPI network and enrichment analyses revealed that *SMURF1* and *SMURF2* are functionally connected with several critical regulators of TGF-β and BMP pathways, including SMAD2/3/5/6/7, TGFBR1/2, RUNX2, and RHOA. These results corroborate previous findings showing that *SMURF1* and *SMURF2* act as E3 ligases that ubiquitinate receptor-regulated SMADs (R-SMADs) and TGF-β receptors, thereby modulating the intensity and duration of TGF-β/BMP signaling [5]. The enrichment of pathways such as “TGF-beta signaling”, “BMP signaling,” and “Wnt signalling pathway” in our network analysis further supports the dual regulatory role of *SMURF1*/*2* in both tumor-suppressive and pro-metastatic mechanisms. The observation that *SMURF1* is downregulated in tumor tissues while high expression correlates with poorer overall survival highlights the multifaceted role of this E3 ligase in breast cancer. This suggests that while *SMURF1* may initially act to suppress early oncogenic signaling (such as Wnt), its presence at higher levels in established tumors could switch to an oncogenic role, promoting more aggressive clinical behavior.

The present study has some limitations. The sample size was relatively small, limiting the power for subtype-specific analyses. Moreover, the mechanistic link between *SMURF1* and Wnt/β-catenin signaling was inferred solely by in silico analysis. Future studies with larger patient cohorts and functional in vitro experiments are warranted to verify these mechanisms.

## 4. Material and Methods

### 4.1. Study Population

The study included 30 newly diagnosed, treatment-naïve breast cancer patients aged between 38 and 82 years. Both tumor and adjacent non-tumorous tissues were collected during mastectomy; normal tissues were obtained from healthy breast tissue surrounding the tumor. The inclusion criterion was a new diagnosis of breast cancer, while patients with carcinoma in situ or bilateral breast cancer were excluded. This study was approved by the Clinical Research Ethics Committee of Muğla Sıtkı Koçman University (Decision No: 240177).

### 4.2. Immunohistochemistry

In this study, a prospective analysis was conducted on tumors diagnosed as invasive breast carcinoma (not otherwise specified) in patients who underwent mastectomy between 2023 and 2024. The study included 30 patients, from whom both tumor and adjacent normal tissue samples were collected from fresh mastectomy specimens. For histopathological examination, tissues were fixed in 10% neutral buffered formalin, embedded in paraffin blocks, and sectioned at 4 μm thickness, followed by hematoxylin and eosin (H&E) staining. After routine microscopic evaluation, the block with the highest tumor content from each case was selected, and 3–4 μm tissue sections were placed on poly-L-lysine-coated glass slides (Thermo Scientific, Waltham, MA, USA) for immunohistochemical (IHC) staining.

### 4.3. RNA Isolation and qPCR

To prevent RNA degradation, tissue samples were preserved in an RNA stabilization reagent (NucleoGene Stabilizer Solution Tissue & Cell DNA/RNA, Cat. No: NGP001) and stored at −80 °C. The collected tissues from each experimental group were then homogenized using a homogenizator (PRO25D Digital Homogenizer). RNA was extracted from breast tissue using the TRIzol™ Reagent (Cat. No: 15596026). cDNA was synthesized using the High-Capacity cDNA Reverse Transcription Kit (ThermoFisher, Cat. No: 4368814). The mRNA levels of *SMURF1* and *SMURF2* were determined with the ABI StepOne Plus instrument using the SYBR Green reagent (NucleoGene, Cat. No: NGMM007). The primer sequences used in this study are summarized in Table 4. *ACTB* was used as the endogenous reference gene, and *SMURF1* and *SMURF2* expression levels were quantified using the 2^−ΔΔCT^ method. The experiments were repeated three times [26].

### 4.4. In Silico Analysis

Our study design and findings were confirmed using several in silico analyses. We used STRING (https://string-db.org/, accessed on 11 November 2025) for the potential interactions and functional enrichment analysis of *SMURF1* and *SMURF2*. The default minimum required interaction score was set to medium confidence (0.400). In addition, STRING reported significant functional enrichment in the pathways associated with these identified interaction networks [27].

#### 4.4.1. Survival Analysis

The prognostic impact of *SMURF1* and *SMURF2* expression was assessed using the Kaplan–Meier Plotter platform (https://kmplot.com/analysis/, accessed on 26 January 2026). Breast cancer patients were stratified into high- and low-expression groups based on the best-performing cutoff expression of each gene. Overall survival (OS) was analyzed using default settings, and hazard ratios (HRs), 95% confidence intervals (CIs), and log-rank *p*-values were recorded.

#### 4.4.2. Differential Expression Analysis

Gene expression levels of *SMURF1* and *SMURF2* in breast tumor tissues and normal breast samples were evaluated using the GEPIA3 web server (https://gepia3.bioinfoliu.com/, accessed on 26 January 2026), which integrates data from the TCGA and GTEx projects. Differential expression was assessed in the TCGA-BRCA cohort.

#### 4.4.3. Single-Cell RNA Sequencing Analysis

To explore the cell-type-specific expression of SMURF genes within the breast tumor, single-cell RNA sequencing datasets were interrogated using the Broad Institute Single Cell Portal (https://singlecell.broadinstitute.org/single_cell, accessed on 26 January 2026). Human breast cancer datasets with annotated cell types were analyzed. *SMURF1* and *SMURF2* expression levels were visualized across major cellular compartments, including malignant epithelial cells, stromal cells, and immune populations. Low-abundance or technically uninformative cell populations were excluded to improve interpretability.

### 4.5. Statistical Analysis

To analyze the obtained gene expression and clinical data, we used R (version 4.12). For paired continuous variables (tumor vs. adjacent normal tissue), the paired *t*-test or Wilcoxon signed-rank test was applied depending on the data distribution. *p*-values < 0.05 were considered statistically significant.

The chi-square test was used for the statistics of categorical variables. The normality of the data distribution was assessed using the Shapiro–Wilk test. The Independent Samples *t*-test was utilized to evaluate statistical differences between groups for continuous variables with a normal distribution. Pairwise comparisons were performed using the Wilcoxon–Mann–Whitney test for continuous variables with a non-normal distribution. For continuous variables, data were summarized as mean ± standard deviation when the distribution was normal, and as median with interquartile range (IQR) when the distribution was non-normal. If the *p*-value was less than 0.05, it was deemed statistically significant.

## 5. Conclusions

This study represents one of the few investigations to comprehensively analyze *SMURF1* and *SMURF2* expression profiles in breast cancer. The results demonstrated a significant reduction in *SMURF1* mRNA levels, suggesting that its downregulation may disrupt the balance of the TGF-β/BMP/Wnt signaling networks, thereby promoting tumor progression. In contrast, the lack of a significant change in *SMURF2* expression indicates that this gene may exert context-dependent functions that vary according to tumor subtype or microenvironmental conditions. The in silico analyses further revealed close interactions of *SMURF1* and *SMURF2* with critical signaling regulators such as SMAD proteins, TGF-β receptors, and RUNX2, emphasizing their importance in maintaining cellular homeostasis and fine-tuning signaling activity. Overall, the decreased *SMURF1* expression observed in breast cancer highlights its potential as a candidate biomarker, whereas the multifaceted role of *SMURF2* warrants deeper investigation. Further functional and translational studies in larger patient cohorts are needed to clarify the roles of these genes across different molecular subtypes of breast cancer and to evaluate their potential as therapeutic targets.

## Figures and Tables

**Table 1:** Distribution of Clinicopathological Variables Among Patients.

Parameters		*n* (%)	*p* *
**Grade**	1	1 (3.3)	<0.001
	2	18 (60.0)	
	3	11 (36.7)	
**ER**	+	26 (86.67)	<0.001
	−	4 (13.3)	
**PR**	+	27 (90)	<0.001
	−	3 (10)	
**cerbB2**	+	3 (10)	<0.001
	−	27 (90)	
**Ki67**	≥14%	15 (50)	>0.05
	<14%	15 (50)	
**Tumor size**	˃2 cm	11 (36.7)	0.003
	≤2 cm	19 (63.3)	
**Age**	˃50	7 (23.3)	0.003
	≤50	23 (76.7)	
**LVI**	+	16 (53.3)	0.72
	−	14 (46.7)	
**PNI**	+	11 (36.7)	0.003
	−	19 (63.3)	
**Lymph node metastasis**	+	17 (56.7)	0.47
	−	13 (43.3)	

* Chi-square test; ER: Estrogen Receptor; PR: Progesterone Receptor; LVI: Lymphovascular invasion; PNI: Perineural invasion.

**Table 2:** Expression levels of *SMURF1* and *SMURF2* in breast cancer and normal tissues.

	Median (IQR)		
Genes	Breast Cancer (*n* = 30)	Normal (*n* = 30)	*p* *
*SMURF1*	0.55 (0.59)	1.16 (1.49)	0.002
*SMURF2*	1.89 (2.03)	1.98 (2.09)	0.981

* Mann–Whitney U test; IQR: Interquartile range.

**Table 3:** Functional enrichments of networks based on *SMURF1* and *SMURF2* interactions.

	Network	Proteins	Network Stats	Database	Description	Strength ^1^	FDR ^2^
1	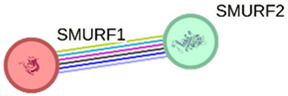	*SMURF1* *SMURF2*	number of nodes: 2 number of edges: 1 average node degree: 1 avg. local clustering coefficient: 1 expected number of edges: 0 PPI enrichment *p*-value: 0.0193	Gene Ontology	GO:0030579 Ubiquitin-dependent SMAD protein catabolic process	3.52	0.0023 0.0348
					GO:0060071 Wnt signaling pathway, planar cell polarity pathway	2.69	
				KEGG Pathways	hsa04340 Hedgehog signaling pathway	2.63	0.0020
2	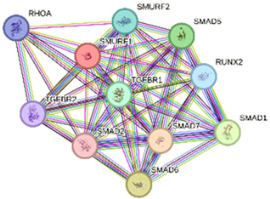	*SMURF1* *SMURF2* SMAD5 RUNX2 SMAD1 SMAD6 SMAD7 SMAD2 TGFBR1 TGFBR2 RHOA	number of nodes: 11 number of edges: 51 average node degree: 9.27 avg. local clustering coefficient:0.941 expected number of edges:13 PPI enrichment *p*-value: 6.66 × 10^−16^	Gene Ontology	GO:0007178 Transmembrane receptor protein serine/threonine kinase signaling pathway	1.94	3.46 × 10^−15^
					GO:0030509 BMP signaling pathway	2.13	1.21 × 10^−10^
				KEGG Pathways	hsa04350 TGF-beta signaling pathway	2.29	2.91 × 10^−20^
3	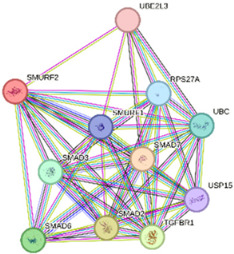	*SMURF2* *SMURF1* SMAD3 SMAD6 SMAD2 TGFBR1 USP15 UBC SMAD7 RPS27A UBE2L3	number of nodes: 11 number of edges: 48 average node degree: 8.73 avg. local clustering coefficient: 0.917 expected number of edges: 17 PPI enrichment *p*-value: 3.4 × 10^−10^	Gene Ontology	GO:0007178 Transmembrane receptor protein serine/threonine kinase signaling pathway	1.84	4.26 × 10^−10^
					GO:0030509 BMP signaling pathway	2.07	4.59 × 10^−8^
				KEGG Pathways	hsa04350 TGF-beta signaling pathway	2.14	6.59 × 10^−12^
	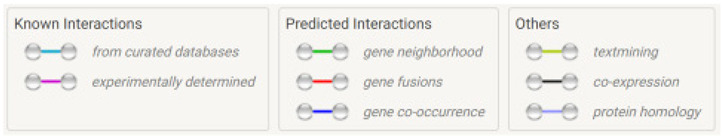		Nodes represent proteins and edges represent protein–protein associations. Colored nodes indicate query proteins and first shell interactors, while white nodes indicate second shell interactors. Edge colors represent different types of interaction evidence, including curated databases, experimental data, gene neighborhood, gene fusion, co-occurrence, text mining, co-expression, and homology.				

^1^ Strength: Log10 (observed/expected). This measure describes how large the enrichment effect is. It is the ratio between (i) the number of proteins in your network that are annotated with a term and (ii) the number of proteins that we expect to be annotated with this term in a random network of the same size. ^2^ False Discovery Rate: This measure describes how significant the enrichment is. Shown are *p*-values corrected for multiple testing within each category using the Benjamini–Hochberg procedure.

**Table 4:** Primer sequences used for qPCR.

Gene/RNA	Primer Sequences
*SMURF1*	F:5′-GTCCAGAAGCTGAAAGTCCTCAGA-3′
	R: 5′-CACGGAATTTCACCATCAGCC-3′
*SMURF2*	F: 5′-GATCCAAAGTGGAATCAGCA-3′
	R: 5′-TGGCATTGGAAAGAAGACG-3′
*ACTB*	F: 5′-CCTGGCACCCAGCACAAT-3′
	R: 5′-GGGCCGGACTCGTCATAC-3′

## Data Availability

All data are available from the corresponding author upon reasonable request.
